# Supplementary material for: Comprehensive Analysis of BR Receptor Expression under Hormone Treatment in the Rubber Tree (Hevea brasiliensis Muell. Arg.)
Source: Plants (Basel). 2023 Mar 11;12(6):1280. doi: 10.3390/plants12061280 (PMC10058276; doi:10.3390/plants12061280)
Supplement: Supplementary file 1 [file plants-12-01280-s001.zip › plants-2233271-supplementary.pdf]

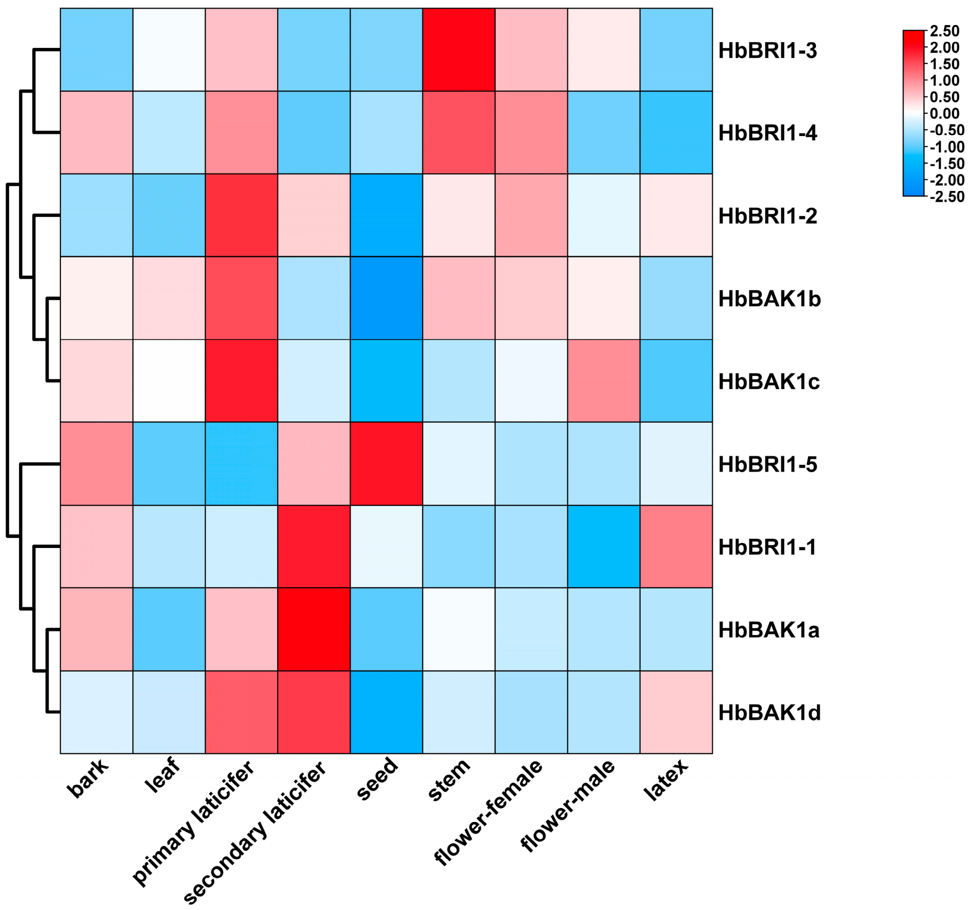

Figure S1: Gene expression level of HbBRI1 and HbBAK1 genes in rubber tree. Heatmap was created by TBtools based on RPKM data. Red and blue indicate high and low expression levels, respectively. Legend on right shows different RPKM values

Table S1 qRT-PCR primers of HbBRI1 and HbBAK1 genes

Gene name	Function	Forward primer (5'-3')	Reverse primer (5'-3')
qHbBRI1-1	qRT-PCR	TGTTGATGCCCGTCGTGTAA	TGGAGGCAGAGAGATCACCA
qHbBRI1-2	qRT-PCR	TGTATGAGTACATGAAGTAT	GTATCCACTGCATTCATGAG
qHbBRI1-3	qRT-PCR	AGCAATCGGATCACAGGCAA	CCAAACCAAGCTGCTGCAAT
qHbBRI1-4	qRT-PCR	TTCTGAGTTGGCAAATGCCTGT	AGCAAAGAACACGAGGAGAAGT
qHbBRI1-5	qRT-PCR	TGGACTACCGATTCCTTTCCA	AGGTCAGCGAGGTGTAGACT
qHbBAK1a	qRT-PCR	TGACTAGCTTGGTGAGCTTGG	CATAGGAATGCGACCCACCA
qHbBAK1b	qRT-PCR	AGCTGGGATCCTACCCTTGT	TTGGCCAGATAGTGCTGCAT
qHbBAK1c	qRT-PCR	AGCGGAACAATTCCGGATGA	TCCCGACAAGCTGTTGTTGT
qHbBAK1d	qRT-PCR	GGTAATGCTGAAGGCGATGC	AGACCCGACAGATTTGCGTT
qHbActin	qRT-PCR	GATGTGGATATCAGGAAGGA	CATACTGCTTGGAGCAAGA
